# Inhibition of IL-13 and IL-13Rα2 Expression by IL-32θ in Human Monocytic Cells Requires PKCδ and STAT3 Association

**DOI:** 10.3390/ijms20081949

**Published:** 2019-04-20

**Authors:** Thu-Huyen Pham, Yesol Bak, Jae-Wook Oh, Jingi Hong, Seungyeoun Lee, Jin Tae Hong, Do-Young Yoon

**Affiliations:** 1 Department of Bioscience and Biotechnology, Konkuk University, Seoul 05029, Korea; huyenpham@konkuk.ac.kr (T.-H.P.); ysbak88@gmail.com (Y.B.); 2 Department of Stem cell and Regenerative Biotechnology, Konkuk University, Seoul 05029, Korea; ohjw@konkuk.ac.kr; 3 Department of Mathematics and Statistics, Sejong University, Seoul 05006, Korea; wlsrl130@gmail.com (J.H.); leesy@sejong.ac.kr (S.L.); 4 College of Pharmacy and Medical Research Center, Chungbuk National University, Chungbuk 28160, Korea

**Keywords:** IL-13Rα2, IL-13 signaling, IL-32θ, STAT3 activation, PKCδ

## Abstract

Interleukin (IL)-32θ, a newly identified IL-32 isoform, has been reported to exert pro-inflammatory effects through the association with protein kinase C delta (PKCδ). In this study, we further examined the effects of IL-32θ on IL-13 and IL-13Rα2 expression and the related mechanism in THP-1 cells. Upon stimulating IL-32θ-expressing and non-expressing cells with phorbol 12-myristate 13-acetate (PMA), the previous microarray analysis showed that IL-13Rα2 and IL-13 mRNA expression were significantly decreased by IL-32θ. The protein expression of these factors was also confirmed to be down-regulated. The nuclear translocation of transcription factors STAT3 and STAT6, which are necessary for IL-13Rα2 and IL-13 promoter activities, was suppressed by IL-32θ. Additionally, a direct association was found between IL-32θ, PKCδ, and signal transducer and activator of transcription 3 (STAT3), but not STAT6, revealing that IL-32θ might act mainly through STAT3 and indirectly affect STAT6. Moreover, the interaction of IL-32θ with STAT3 requires PKCδ, since blocking PKCδ activity eliminated the interaction and consequently limited the inhibitory effect of IL-32θ on STAT3 activity. Interfering with STAT3 or STAT6 binding by decoy oligodeoxynucleotides (ODNs) identified that IL-32θ had additive effects with the STAT3 decoy ODN to suppress IL-13 and IL-13Rα2 mRNA expression. Taken together, our data demonstrate the intracellular interaction of IL-32θ, PKCδ, and STAT3 to regulate IL-13 and IL-13Rα2 synthesis, supporting the role of IL-32θ as an inflammatory modulator.

## 1. Introduction

Interleukin (IL) IL-13 is produced largely in Th2 lymphocytes and is implicated in the pathogenesis of allergic disorders [1,2]. The biological activities of IL-13 are modulated through a receptor complex composed of the IL-4 receptor alpha (Rα) associated with the IL-13Rα1 chain [3]. IL-13 can utilize both IL-4Rα/JAK2/STAT3 and IL-13Rα1/TYK2/STAT1/STAT6 signaling pathways to regulate the expression of some critical inflammatory genes [4]. In addition, IL-13 receptor alpha subunit 2 (IL-13Rα2), a unique receptor of IL-13, has recently been reported to contribute to signaling, which is distinct from its original definition as a decoy receptor of IL-13 due to the lack of region for signal transduction [5]. A low level of IL-13Rα2 mRNA expression has been detected in promonocytic U937 cells and fibroblasts [3]. It may function alone or heterodimerize with an unknown receptor to recruit MAPK signaling factors, therefore competing with IL-13Rα1 for IL-13 binding [6]. It has been suggested that IL-13Rα2 activated by IL-13 and TNF-α could further be involved in IL-13 signaling to stimulate activator protein 1 (AP-1) containing c-jun and fra-2 to induce transforming growth factor beta 1 (TGF-β1) production in macrophages [7]. Another study determined that IL-13Rα2 may form a heterodimeric complex with the chitinase-like protein family member, Chi3l1, and that IL-13 can use this complex to induce TGF-beta production [8]. These studies suggest that IL-13Rα2 has dual functions, including a decoy receptor function and signal transduction, depending on its relative expression and the source of IL-13. 

The exact mechanisms by which IL-13Rα2 mediates the activation of downstream signaling pathways remain unclear. STAT6 cannot be activated through IL-13Rα2 because IL-13Rα2 has a short cytoplasmic tail that does not bind to JAKs or STATs [5,9,10], even though STAT6 plays critical role in the transcriptional activation of IL-13Rα2 [3]. Alternatively, STAT3 can be activated through IL-13Rα2, and the activation of STAT3 mediated by IL-13Rα2 does not require a direct physical interaction [11]. Moreover, STAT3 can be activated directly through IL-13Rα1, which contains tyrosine residues and might serve as docking sites for STAT3 [12,13]. It is well-known that IL-13 can induce STAT6 signaling [2], and recent studies have also reported that IL-13 could activate STAT3 signaling [4,14]. Taken together, alongside the activation of STAT6, the activation of STAT3 might play a major role in IL-13 and IL-13Rα2 signaling. 

STAT3, a transcription factor belonging to the STAT family of transcription factors, can be phosphorylated on both Tyr705 and Ser727 residues [15,16]. While STAT3 tyrosine phosphorylation is essential for dimerization, nuclear translocation, and binding to the DNA-response elements of target genes [15,17,18], the serine phosphorylation involves signal transduction and its role in STAT3’s transcriptional activity remains controversial [19,20,21,22]. Protein kinase C-δ (PKCδ), a member of the novel protein kinase C (PKCs) (δ, ε, η, θ) subclass [23], is a source of serine/threonine kinase that is responsible for the phosphorylation and signal transduction of various factors including STAT3 [24,25,26,27,28]. Several lines of evidence have shown that PKCδ specifically associated with STAT3 and phosphorylated STAT3 on Ser727, leading to an inhibition of STAT3 DNA binding and transcriptional activity [25,28,29]. This kinase was found to be activated by IL-13, and inhibition of PKCδ activation greatly suppressed IL-13 induced STAT3 DNA binding [14,29]. Collectively, these studies support the involvement of PKCδ and STAT3 in IL-13 and IL-13Rα2 downstream signaling.

IL-32θ, a recently identified IL-32 isoform [30], has been reported to exert an inhibitory effect on CCL5 expression through its interaction with PKCδ and STAT3 in THP-1 monocytic cells [31]. Other functional effects of IL-32θ on inflammatory cytokines, including TNF-α and IL-1β, were also affirmed to be related to PKCδ [32,33], suggesting that this interaction could be further explored to investigate other effects of IL-32θ on inflammatory responses. In this study, we attempted to discover whether the known association between IL-32θ, PKCδ, and STAT3 would disturb IL-13Rα2 and IL-13 expression in phorbol 12-myristate 13-acetate (PMA)-stimulated THP-1 cells. Our data indicated that the inhibition of IL-32θ on STAT3 activation through PKCδ led to a decrease of STAT3 DNA binding to IL-13 and IL-13Rα2 promoters and thus down-regulated their expression.

## 2. Results

### 2.1. Microarray Identification of IL-13Rα2 and IL-13 as Down-Regulated Genes by IL-32θ

The THP-1 monocytic cell lines that constitutively express IL-32θ (THP-1/IL-32θ) or an empty vector (THP-1/EV) were used to perform microarray analysis upon PMA stimulation. Of the IL-32θ-down-regulated genes, CCL5 was previously identified as being regulated by IL-32θ, and the mechanism was proven to be through the PKCδ and STAT3 association [31]. In this study, we further explored the previous microarray profile [31] and found that IL-13Rα2 was remarkably reduced in THP-1/IL-32θ when compared to THP-1/EV (Table 1). In addition, IL-13 expression was slightly decreased by IL-32θ, suggesting that IL-32θ may have effects on IL-13 signaling through IL-13Rα2.

### 2.2. IL-32θ Negatively Regulates IL-13Rα2 and IL-13 in Both mRNA and Protein Expression

**Figure 1:**
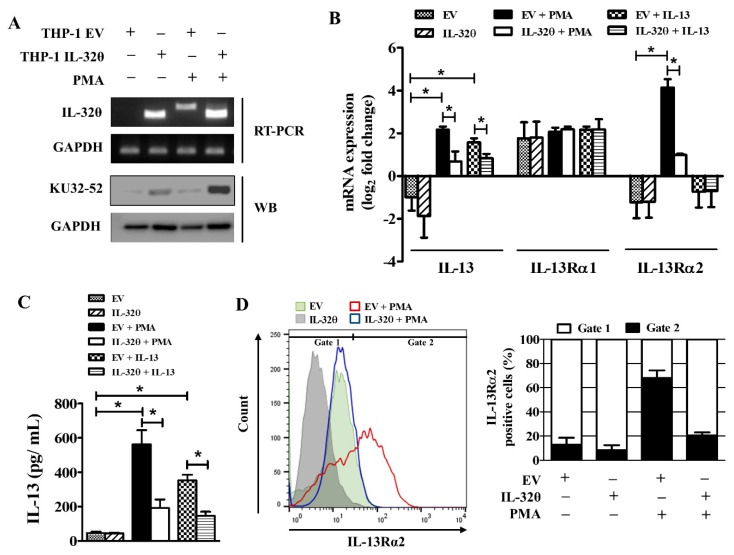
IL-32θ inhibits IL-13 and IL-13Rα2 expression. (**A**) Constitutive expression of IL-32θ in stably expressing THP-1 cell model was confirmed by RT-PCR and Western blotting analyses. (**B**) mRNA expression of IL-13, IL-13Rα1, and IL-13Rα2 in THP-1/EV (empty vector) or THP-1/IL-32θ upon phorbol 12-myristate 13-acetate (PMA) (10 nM) or IL-13 (20 ng/ mL) stimulation after 24 h (*n* = 4). (**C**) Secretion level of IL-13 after PMA treatment for 24 h was measured by ELISA (*n* = 3). (**D**) Flow cytometry analysis was performed to assess the cell surface expression of IL-13Rα2 by using the anti-human IL-13Rα2 antibody. Data are shown as mean ± SEM. Statistical significance was analyzed using two-way ANOVA test followed by multiple comparison tests (* *p* < 0.05). Results are representative of single experiments.

The expression of IL-32θ in THP-1/IL-32θ cells can be recognized by a specific primer set, as described in [32], based on the size of the amplicons, such as 299 bp for IL-32θ and 360 bp for other IL-32 isoforms. The protein expression of IL-32θ was also confirmed by Western blot using the KU32-52 antibody (Figure 1A). To validate the function of IL-32θ on IL-13Rα2 and IL-13 signaling, we measured the mRNA and the protein expression of those factors. It was observed that the mRNA expression of both IL-13 and IL-13Rα2 was reduced significantly by IL-32θ upon PMA stimulation (Figure 1B). The expression of IL-13Rα1, which was constitutively expressed in the monocytes, was also confirmed to remain unchanged and was not the target of IL-32θ (Figure 1B). As IL-13Rα2 is known as a decoy receptor of IL-13, we treated both THP-1 cell lines with IL-13 recombinant protein to confirm this event. It was found that IL-13 could activate IL-13 mRNA expression but not IL-13Rα2 expression, and the IL-13 activation by IL-13 was inhibited by IL-32θ (Figure 1B). A similar pattern was seen in the IL-13 secretion levels upon PMA or IL-13 stimulation (Figure 1C). IL-13Rα2 was shown to exist as an intracellular molecule, and after treatment of cells with the stimulator, it was rapidly mobilized to the cell surface [34]. Flow cytometry analysis indicated that IL-13Rα2 intensity was increased on the cell surface of the PMA-stimulated THP-1/EV, whereas this level was dramatically decreased in THP-1/IL-32θ (Figure 1D). These results demonstrate that IL-32θ has negative regulatory effects on IL-13Rα2 and IL-13 expression. 

### 2.3. IL-32θ Directly Interacts with PKCδ, STAT3, but not STAT6

**Figure 2:**
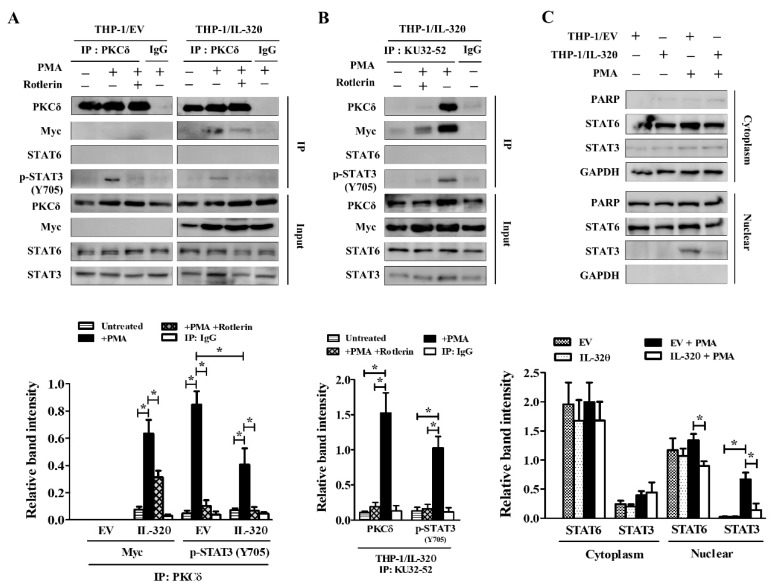
Interaction between IL-32θ, PKCδ, STAT3, and STAT6 under PMA stimulation. Cells were treated with 10 nM PMA for 24 h before lysate. Immunoprecipitated proteins and the input were probed with the indicated antibodies as visualized by Western blotting. (**A**) Endogenous PKCδ was immunoprecipitated from THP-1/EV and THP-1/ IL-32θ cells. (**B**) IL-32 antibody KU-32-52 was used for the immunoprecipitation of IL-32θ in THP-1/IL-32θ cells. (**C**) Cells were treated with 10 nM PMA for 1 h before performing nuclear and cytoplasm fractionation followed by Western blot analysis. Data are shown as mean ± SEM (*n* = 3). Statistical significance was analyzed using two-way ANOVA test followed by multiple comparison tests (* *p* < 0.05). Western blot bands were quantified by Fiji software. Results are representative of single experiments.

The association of PKCδ, STAT3, and IL-32θ is responsible for IL-32θ’s inhibitory effect on CCL5 expression [31]. Furthermore, STAT6, another STAT molecule, is a critical transcription factor of IL-13 signaling, which can initiate the transcription of various downstream inflammatory genes [1,35]. Thus, the relationship between PKCδ, STAT3, STAT6, and IL-32θ should be considered to investigate the mechanism by which IL-32θ is prompted to inhibit IL-13 signaling. To clarify this issue, we performed immunoprecipitation analysis on THP-1/EV and THP-1/IL-32θ upon PMA stimulation. The result specified that PMA-activated endogenous PKCδ directly interacted with IL-32θ and STAT3 at the phosphorylation of Tyrosine705, but not STAT6 (Figure 2A). Using the IL-32 monoclonal antibody KU-32-52 to precipitate IL-32θ and its related factors in THP-1/IL32θ, we found that IL-32θ also interacted with STAT3 and PKCδ, but not STAT6 (Figure 2B). The interaction between IL-32θ and STAT3 required PKCδ because blocking PKCδ activation by rottlerin, a specific PKCδ inhibitor at low concentrations [36], removed the inhibitory effect of IL-32θ on STAT3 tyrosine phosphorylation (Figure 2A,B). To prove that IL-32θ reduced the transcriptional activity of STAT3, we examined the nuclear translocation of STAT3 visualized by Western blot analysis. After treatment with PMA for 1 h, the level of STAT3 increased quickly in the nucleus of THP-1/EV cells, while it was lower in the case of the THP-1/IL-32θ cells. The nuclear translocation of STAT6, which seemed to remain steady and not be affected much by PMA activation, was also abrogated by IL-32θ (Figure 2C). Overall, these data indicated that although STAT6 activity in response to IL-13 signaling has been well-documented, it might not belong to the molecular mechanisms whereby IL-32θ inhibits IL-13 and IL-13Rα2 expression. Instead, activation of STAT3, which could be involved in IL-13 signaling through IL-13Rα2, was the target of IL-32θ through the association with PKCδ.

### 2.4. STAT3 Binding to IL-13Rα2 and IL-13 Promoters Was Suppressed by IL-32θ

**Figure 3:**
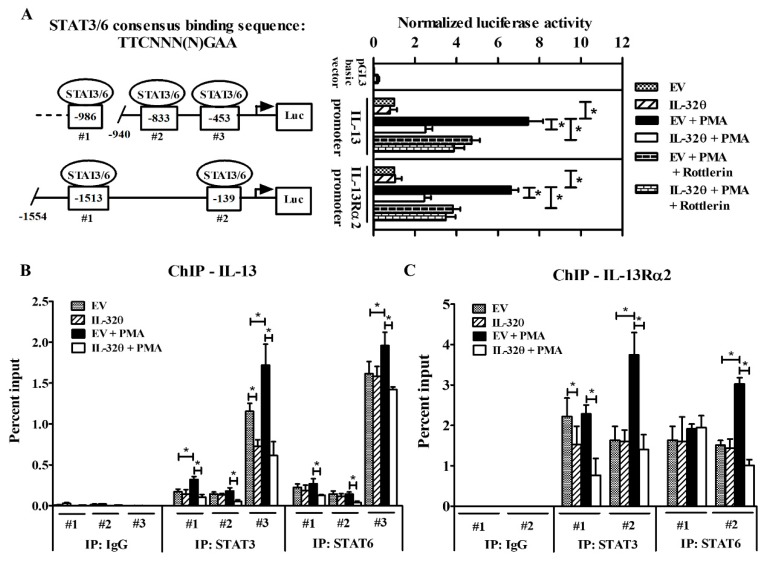
Promoter analysis of IL-13 and IL-13Rα2 containing STAT3 and STAT6 binding sites. (**A**) Left panel, diagram showing putative element sites for STAT3 or STAT6 binding to promoters. Right panel, the luciferase assay was analyzed after transfecting IL-13 and IL-13Rα2 promoter reporter plasmids to THP-1/EV or THP-1/IL-32θ cells followed by 10 nM PMA treatment (*n* = 4). (**B**,**C**) Chromatin immunoprecipitation (ChIP) assay was performed by using the DNA eluted from the immunoprecipitation of STAT3 and STAT6. Quantitative PCR results (*n* = 3) were obtained by running primers designed to target three binding sites of STAT3 or STAT6 on the IL-13 promoter (**B**) and two binding sites on the IL-13Rα2 promoter (**C**). Data are shown as mean ± SEM. Statistical significance was analyzed using two-way ANOVA test followed by multiple comparison tests (* *p* < 0.05). Results are representative of single experiments.

To identify whether IL-32θ could affect the binding of STAT3 to IL-13 and IL-13Rα2 promoters, we sought the promoter regions of both genes that contained the common STAT3 or STAT6 consensus binding sequence TTCNNN(N)GAA [31,37,38] to construct reporter vectors and measure promoter activities through the luciferase assay. We selected three potential binding sites, (−453 to −445), (−833 to −825), and (−986 to −977), for STAT3/6 in IL-13 promoter, and two binding sites, (−1513 to −1504) and (−139 to −130), in IL-13Rα2 promoters. Luciferase results showed that IL-13 and IL-13Rα2 promoters, which were significantly activated under PMA treatment in THP-1/EV, were suppressed in THP-1/IL-32θ cells (Figure 3A). Furthermore, blocking PKCδ activity by treating cells with rottlerin prior to PMA stimulation reduced the promoter activities of IL-13 and IL-13Rα2 in both the THP-1/EV and the THP-1/IL-32θ cells (Figure 3A). This finding is in line with a previous study that PKCδ was a negative regulator for STAT3 DNA binding [31]. Thus, PKCδ exclusion impaired the STAT3-PKCδ complex followed by a decrease of STAT3 binding activity, which might be necessary for IL-13 and IL-13Rα2 transcription. Notably, IL-32θ and PKCδ exerted additional inhibitory effects on the STAT3 transcription activity (Figure 3A).

Since STAT transcription factors may bind to similar sequences, the next question is whether the STAT6 is also involved in IL-13 and IL-13Rα2 promoter activation suppressed by IL-32θ. Therefore, we performed chromatin immunoprecipitation (ChIP) assay to explore this issue. It was observed that STAT3 and STAT6 preferred the proximal binding site (−453 to −445) to initiate IL-13 promoter (Figure 3B) and the (−139 to −130) site to increase IL-13Rα2 promoter (Figure 3C). While IL-32θ repressed almost all STAT3 binding sites to IL-13 or IL-13Rα2 promoters upon treatment or non-treatment with PMA, it affected STAT6 sites only in the PMA-stimulated condition. Taken together, these data suggest that IL-32θ constitutively targeted STAT3 DNA binding to mediate IL-13 or IL-13Rα2 promoter activities and partly interfered with STAT6 DNA binding in PMA-stimulated cells. 

### 2.5. Blocking of STAT3 Binding Showed Additive Effect with IL-32θ

**Figure 4:**
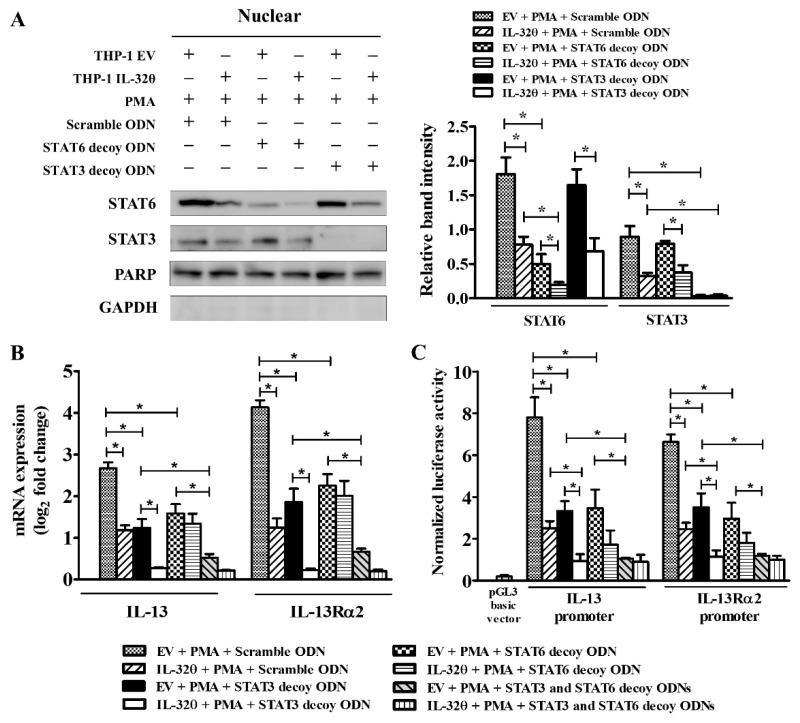
Blocking STAT3 or STAT6 binding activities by decoy oligodeoxynucleotides (ODNs). THP-1/EV and THP-1/IL-32θ cells were transfected with STAT3 or STAT6 or scrambled decoy ODNs for 24h followed by treatment with PMA (10 nM) for 1 h before being prepared for further analyses. (**A**) Western blot result of the nuclear protein probed with anti-STAT3 or STAT6 antibodies (*n* = 3). (**B**) mRNA expression of IL-13 and IL-13Rα2 conducted by RT-qPCR (*n* = 3). (**C**) Relative luciferase activities of IL-13 and IL-13Rα2 promoters (*n* = 4). Data are shown as mean ± SEM. Statistical significance was analyzed using two-way ANOVA test followed by multiple comparison tests (* *p* < 0.05). Western blot bands were quantified by Fiji software. Results are representative of single experiments.

To investigate whether IL-32θ would regulate IL-13 and IL-13Rα2 expression via STAT3 or STAT6 binding, we used STAT3 or STAT6 decoy oligodeoxynucleotides (ODNs) to prevent the transcriptional activities of STAT3 or STAT6 separately in THP-1/EV and THP-1/IL-32θ cells. Accordingly, the amount of STAT6 in the nucleus was decreased significantly, and nuclear STAT3 accumulation was totally abolished (Figure 4A). In addition, transfection of either STAT3 or STAT6 decoy ODNs dramatically reduced PMA-induced IL-13 and IL-13Rα2 mRNA expression and luciferase activities, suggesting that both factors played roles in the transcription of these genes (Figure 4B,C). Importantly, IL-32θ collaborated with the STAT3 decoy ODN to down-regulate IL-13 and IL-13Rα2 mRNA expression when compared to the scrambled decoy transfected cells. However, no additive effect was seen in the case of STAT6 decoy ODN transfection (Figure 4B,C). Moreover, co-treatment with both STAT3 and STAT6 decoy ODNs almost eliminated the mRNA expression and the luciferase activities of IL-13 and IL-13Rα2 (Figure 4B,C). Thus, it can be inferred that the addictive effect of IL-32θ on the STAT3 decoy ODN that disrupted PMA-induced IL-13 and IL-13Rα2 expression could result from direct interaction with STAT3.

## 3. Discussion

Our previous studies have reported that IL-32θ has modulating effects on pro-inflammatory cytokines and chemokines in human monocytic THP-1 cells [31,32,33]. When screening potential genes regulated by IL-32θ based on our previous microarray data [31], we found that IL-13Rα2 and IL-13 expression were notably down-regulated in THP-1/IL-32θ cells. This raised the question of whether IL-32θ regulates IL-13 signaling through IL-13Rα2 and by which mechanism it might act. We therefore performed different experiments to clarify this issue.

It is well-known that IL-13Rα2 is a decoy receptor for IL-13 that can bind to IL-13 and does not activate STAT6 signal transduction [5]. The expression of IL-13Rα2 requires STAT6 and NF-κB transcriptional activation and can be stimulated by IL-13 or IL-4 in combination with TNF-α (but not in any of these cytokines alone) in THP-1 cells [7]. It was found here that PMA could activate both IL-13 and IL-13Rα2 expression. PMA is a non-specific stimulator that can increase kinase phosphorylation followed by STAT proteins or NF-κB activation [39,40,41]. PMA can also activate T_H_2 cytokines including IL-13 and IL-4 [42], therefore, it is useful as an inducer for IL-13 and IL-13Rα2 expression. Currently, we have identified that IL-32θ inhibits IL-13 and IL-13Rα2 transcription and protein expression upon PMA stimulation in THP-1 cells. 

When IL-13 recombinant protein was used to trigger IL-13 signaling without stimulating IL-13Rα2, we found that IL-13 expression was also reduced by IL-32θ. This result suggests that IL-32θ might target STAT6, which is an important transcription factor in most functions of IL-13 and is crucial for IL-13Rα2 transcription, even though IL-13Rα2 cannot transduce signals through STAT6. Another possible mechanism could be the connection between IL-32θ and PKCδ, which was proved to be essential to regulate IL-1β, TNF-α and CCL5 products [31,32,33]. Both PMA and IL-13 are sources for PKCδ activation [14,39]. Unfortunately, we could not find any direct interaction between PKCδ and STAT6; however, the association between IL-32θ, endogenous PKCδ, and STAT3 was confirmed in PMA-stimulated THP-1 cells. Our prior study demonstrated that IL-32θ acted as a bridge to form a trimeric complex with PKCδ, and STAT3 then facilitated the phosphorylation of STAT3 on Ser727 and consequently inhibited STAT3 activity [31]. This finding is in line with other studies where PKCδ preferentially associated with the Tyr705-phosphorylated-STAT3 but phosphorylated STAT3 on Ser727 instead of Tyr705 and thereby blocked STAT3 nuclear translocation [28]. Since our previous study also determined that IL-32θ directly interacted with STAT3 Ser727, but not Tyr705 [31], we therefore blocked PKCδ activity by using rottlerin upon PMA stimulation to assess whether IL-32θ still interacted with activated STAT3 without PKCδ. However, the requirement of PKCδ for IL-32θ and STAT3 coherence is indispensable, since the removal of PKCδ totally eliminated this interaction and restricted IL-32θ’s inhibitory effects on IL-13 and IL-13Rα2 promoters. Based on these points, we further examined STAT3 transcriptional activity on the regulation of IL-13 and IL-13Rα2 expression. We found that there were three potential binding sites for STAT3 or STAT6 in the IL-13 promoter and two sites in the IL-13Rα2 promoter. By conducting the ChIP assay, we determined that STAT3 and STAT6 bound to the same sequences on the IL-13 and IL-13Rα2 promoter and both preferred the proximal sites for promoter activities. Interestingly, IL-32θ constitutively inhibited STAT3 DNA binding, while it only affected the stimulated STAT6. This result suggested that STAT3 might be a key point for IL-32θ to act through, while STAT6 was partly involved in the IL-32θ mediated mechanism.

It was observed that the transcriptional activity of STAT3 or STAT6 is necessary for IL-13 expression stimulated either by IL-13 or PMA; however, the activation of IL-13Rα2 requires more transcription factors than only STAT3 or STAT6 activity. From studies providing evidence that TNF-α can synergize with IL-13 to regulate the IL-13Rα2 promoter [3], and STAT6 plus NF-κB are essential for IL-13Rα2 activation [7], it can be inferred that IL-13 was the source for STAT6 activation, while TNF-α was the source for NF-κB activation to induce IL-13Rα2 expression. Our previous study verified that IL-32θ targeted PMA-induced NF-κB to inhibit TNF-α products [33]. Thus, the next question was whether IL-32θ would target NF-κB together with STAT3 or STAT6 to regulate IL-13Rα2. Hence, we performed the ChIP assay to analyze p65, an NF-κB subunit, binding to IL-13 and IL-13Rα2 promoters, but the result showed that p65 was not involved in the IL-32θ-regulated mechanism on IL-13 and IL-13Rα2 expression (Supplementary Figure S1). 

As indicated above, although IL-32θ and STAT6 had no interaction, the binding activity of STAT6 to IL-13 and IL-13Rα2 promoters was still disrupted by IL-32θ. Furthermore, no additive effect was seen between IL-32θ and the STAT6 decoy ODN to regulate IL-13 and IL-13Rα2 expression. It can be assumed that IL-32θ regulated STAT6 DNA binding activity via an indirect way. IL-32θ could reduce IL-13 expression by prohibiting STAT3 activation through PKCδ, then the lack of newborn IL-13 expression might not trigger STAT6 activity in THP-1/ IL-32θ as compared to that in THP-1/EV. Further study is required to explore this repression mechanism. 

**Figure 5:**
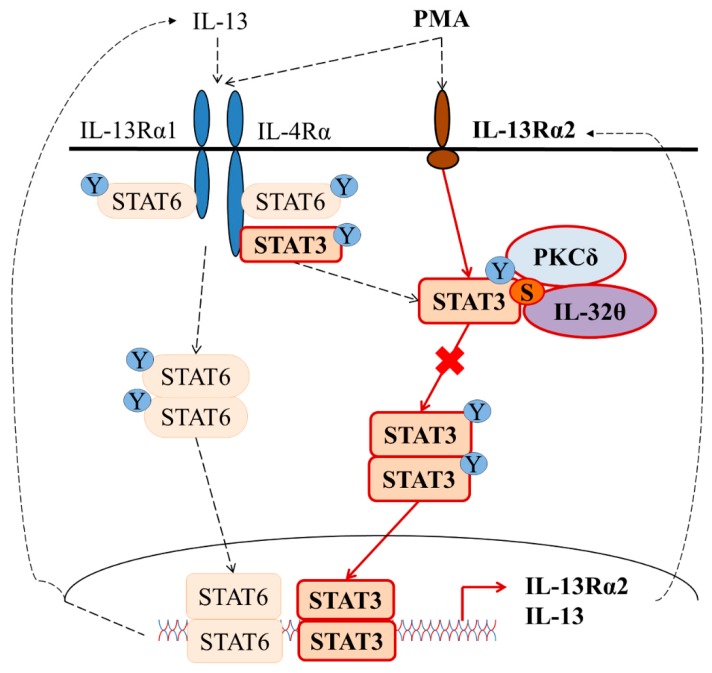
Signaling pathways for the IL-32θ-mediated down-regulation of IL-13 and IL-13Rα2 expression in THP-1 cells. Briefly, IL-13 stimulation causes the activation of both STAT3 and STAT6 to regulate IL-13 expression, but not IL-13Rα2, while PMA stimulation can activate both transcription factors and trigger IL-13 and IL-13Rα2 expression. IL-32θ suppresses IL-13 and IL-13Rα2 by interacting directly with PKCδ and STAT3 and subsequently reduces STAT3 binding activity to promoters. STAT6 activity may be partly and indirectly disturbed by IL-32θ. “Y” stands for Tyrosine. “S” stands for Serine.

In summary, we discovered here that IL-32θ bound to PKCδ and supported PKCδ to inhibit STAT3 DNA binding activity followed by a reduction of IL-13 and IL-13Rα2 synthesis. Figure 5 shows a schematic diagram of this proposed mechanism. This study advances our understanding of IL-32θ as an intracellular modulator through PKCδ and STAT3. Since IL-13 signaling and its unique receptor IL-13Rα2 contribute to the prolongation of inflammation in various infectious diseases, this study might be useful in seeking potential therapeutic targets to prevent Th2 cytokine-related diseases. 

## 4. Materials and Methods.

### 4.1. Cell Culture and Generation of IL-32θ-Stably Expressing Cell Line

The human monocytic cell line THP-1 (Korean Cell Line Bank, Seoul, Korea, KCLB-40202) was cultured in RPMI-1640 (Hyclone, Logan, UT, USA) supplemented with 10% heat-inactivated fetal bovine serum (FBS) (MilliporeSigma, Burlington, MA, USA), 100 units/mL penicillin, and 100 μg/mL streptomycin. The IL-32θ stably expressing THP-1 cell line was generated as previously described in [32].

### 4.2. Microarray

The list of gene expression patterns by microarray between IL-32θ stably expressing THP-1 cells and empty vector THP-1 cells were extracted from previous experiments [31]. The signal value was calculated as the ratio between IL-32θ expressed cells and empty vector cells and then converted to log_2_ values. 

### 4.3. Reverse Transcription Quantitative PCR (RT-qPCR)

mRNA expression levels in the THP-1 cells were detected by RT-qPCR. Total RNA from these cells was isolated using Easy-BLUE (iNtRON Biotechnology, SungNam, Korea), then the reverse transcription was performed. RT-PCR was conducted with a relative quantification protocol using SensiFAST^™^ SYBR NO-ROX Kit (BIOLINE, London, UK) and Rotor-Gene 6000 series software 1.7 (QIAGEN, Venlo, The Netherlands). Table 2 shows the primer sets that were used to analyze the samples. Transcript levels were calculated based on the ΔΔ*C*t method [43]. The mRNA expression was presented as log_2_ of fold change. 

### 4.4. Nuclear and Cytoplasmic Fractionation

THP-1 cells (1 × 10^6^ cells/mL) were stimulated with PMA (Millipore Sigma, Burlington, MA, USA) 10 nM for the indicated time. Cells were collected and fractionated using the NE-PER Nuclear and Cytoplasmic Extraction kit (Thermo Fisher Scientific, Waltham, MA, USA), according to the manufacturer’s protocol.

### 4.5. Immunoprecipitation and Western Blotting

Cell lysates were prepared in radioimmunoprecipitation assay (RIPA) buffer (DyneBio, Seoul, Korea) containing 1× complete protease inhibitor cocktail and 1× PhoSTOP (Roche Diagnostics, Mannheim, Germany). For immunoprecipitation, 1 mg of the cell lysates were mixed with 1 μg of antibody and then pulled down using 40 μL of protein G-agarose beads (KPL, Gaithersburg, MD, USA). A portion of whole cell lysates (50 μg) was used as the input prior to the immunoprecipitation. Samples were subjected to 10% SDS–PAGE before being transferred to polyvinylidene difluoride (PVDF) membranes (Millipore Sigma). The membranes were hybridized with appropriate primary antibodies at 4 °C overnight. Western blotting was developed by a chemiluminescence detection kit (Advanstar, Cleveland, OH, USA) and detected using the EZ-capture MG protein imaging system (ATTO, Tokyo, Japan). An antibody specific to Myc-tag was purchased from Millipore Sigma. Antibodies specific to STAT6 and Tyr705 phosphorylated STAT3 were from Novus (Littleton, CO, USA). The monoclonal antibody KU-32-52 was prepared as previously reported in [44]. Other antibodies were from Cell Signaling Technology (Danvers, MA, USA). Western blot bands were quantified by Fiji, an ImageJ open-source software [45]. The relative band intensity was calculated as a ratio as follows:

$$\frac{\mathrm{Intensity}\;\left(\mathrm{Target}\right)/\;\mathrm{Intensity}\;\left(\mathrm{Background}\;\mathrm{of}\;\mathrm{target}\right)}{\mathrm{Intensity}\;\left(\mathrm{Control}\right)/\;\mathrm{Intensity}\;\left(\mathrm{Background}\;\mathrm{of}\;\mathrm{control}\right)}$$

### 4.6. Enzyme-Linked Immunosorbent Assay (ELISA)

The cell culture supernatants were analyzed using the human IL-13 ELISA kits (R&D Systems, Minneapolis, MN, USA), according to the manufacturer’s protocol. 

### 4.7. Decoy Oligodeoxynucleotides (ODNs) and Transfection

Single-stranded ODN sequences were synthesized as previously described [7,46]: STAT3 decoy ODN, 5′-CATTTCCCGTAAATC-3′ and 5′-GATTTACGGGAAATG-3′; STAT6 decoy ODN, 5′-GAT CAAGACCTTTTCCCAAGAAATCTAT-3′ and 5′-ATAGATTTCTTGGGAAAGGTCTTGATC-3′; scrambled decoy ODN, 5′-CATGTCGTCACTGCGCTCAT-3′ and 5′-ATGAGCGCAGTGACGACA TG-3′. Double-stranded ODNs were prepared in annealing buffer (10 mM Tris–HCl, 1 mM EDTA, pH 8.0) by melting at 95 °C for 15 min, then cooled to room temperature (rt) for 2 h. To block STAT3 or STAT6 signaling, THP-1 cells (1 × 10^6^/mL) were transfected with 200 nM ODNs by using Lipofectamine 2000 (Invitrogen, Carlsbad, CA, USA) for 24 h prior to the addition of PMA.

### 4.8. Plasmid Constructs

The promoter fragments (−1574 to +395) for IL-13RΑ2 gene encoding for IL-13Rα2 protein were amplified by using these primers: 5′-tcccccgggTGGGTTAGTGGCATTGAAAG-3′ (forward), 5′-ccgctcgagACCATCACCAAA-AAAACCAG-3′ (reverse). The PCR products containing restriction sites for *Sma*I and *Xho*I were digested and inserted into the pGL3 basic vector (Promega Corporation, Madison, WI, USA). The IL-13 promoter (−940 to +48) luciferase vector was purchased from Addgene (Cambridge, MA, USA).

### 4.9. Luciferase Assay

Cells were seeded in 6-well plates at a density of 1 × 10^6^ cells/ mL and then co-transfected with the IL-13 or IL-13Rα2 promoter (1 μg/mL) and pRL-null Renilla luciferase vector (0.1 μg/mL) as the control using Lipofectamine 2000 (Invitrogen). After 24 h incubation, cells were washed with phosphate buffered saline (PBS) to remove the transfection buffer, and 1 × 10^6^ cells/mL was counted for treatment with PMA (10 nM) for a further 24 h. Cells were collected, and protein was extracted using the Luciferase Reporter Assay System (Promega Corporation). Firefly luciferase activities were measured by VICTOR X3 (PerkinElmer Inc., Waltham, MA, USA) and normalized to *Renilla* luciferase activities. The pGL3 basic vector (Promega), which does not contain a eukaryotic promoter sequence, was used as a negative control. 

### 4.10. Chromatin Immunoprecipitation (ChIP) Assay

The ChIP assay was assessed using the Simple ChIP Enzymatic Chromatin IP kit (Cell Signaling Technology) according to the manufacturer’s instructions. Briefly, proteins were cross-linked with DNA, then the protein/DNA complexes were precipitated by an anti-STAT3 antibody or anti-STAT6 antibody or anti-IgG (cell signaling technology) as the control. After collection with Protein G Agarose, the eluted DNA was amplified by qPCR using designed primers for STAT3/6 or NF-κB binding sites in the IL-13 (NG_012090.1) or IL-13Rα2 (NG_012514.1) promoters (Table 2). Data are presented as the intensity of immunoprecipitated DNA versus 2% input DNA.

### 4.11. Flow Cytometry Analysis

Cells were stimulated with 10 nM PMA for 24 h before being collected and incubated with goat anti-human polyclonal IL-13Rα2 antibody (R&D Systems) for 30 min at 4 °C. After washing with PBS, rabbit anti-goat IgG antibody rhodamine conjugate (Millipore Sigma) was incubated for a further 15 min. Fluorescence was measured by FACSCalibur (BD, Franklin Lakes, NJ, USA) and analyzed by FlowJo software (Tree Star Inc., Ashland, OR, USA). Normal IgG antibody (Santa Cruz Biotechnology Inc., Santa Cruz, CA, USA) was used as the isotype control for gating. The gating strategy is explained in detail in Supplementary Figure S2. 

### 4.12. Statistics

All experiments were repeated at least three times, and data are presented as mean ± standard error of the mean (SEM). A two-way ANOVA analysis was performed under the normality assumption, because the residual plots showed the goodness of fit for the normal distribution. To evaluate the difference of gene or protein expression within the THP-1/IL-32θ and the THP-1/EV cell lines under different treatment conditions, pairwise t-tests were conducted with Dunnett’s multiple comparison adjustment, because the comparison of the control versus the other treatments was of interest. If all possible pairwise comparisons were of interest, Tukey’s multiple comparison adjustment was applied. The statistical analyses were performed using GraphPad Prism software version 5.0 and R program version 3.5.1. All *p*-values were two-sided, and *p* < 0.05 was defined as being statistically significant.

## Figures and Tables

**Table 1:** List of Interleukin (IL)-32θ down-regulated genes identified by microarray analysis.

Gene Accession	Gene Description	Gene Symbol	Ratio Value (log_2_)	GO Biological Process Term
Down-regulation				
NM_005988	Small proline-rich protein 2A	SPRR2A	−3.866	Epidermis development // keratinocyte differentiation
NM_002985	Chemokine (C-C motif) ligand 5	CCL5	−2.876	Chemotaxis // inflammatory response // immune response
NM_001712	Carcinoembryonic antigen-related cell adhesion molecule 1	CEACAM1	−2.612	Angiogenesis // integrin-mediated signaling pathway // cell migration
**NM_000640**	**Interleukin 13 receptor, alpha 2**	**IL13RA2**	**−2.399**	**---**
NM_002160	Tenascin C	TNC	−2.314	Cell adhesion // signal transduction // response to wounding
NM_005546	Interleukin 2-inducible T-cell kinase	ITK	−3.250	Cellular defense response // signal transduction // intracellular signaling pathway
NR_029635	MicroRNA 221	MIR221	−2.242	---
NM_001558	Interleukin 10 receptor, alpha	IL10RA	−1.526	Blood coagulation // response to lipopolysaccharide
NM_000201	Intercellular adhesion molecule 1	ICAM1	−1.231	Regulation of cell adhesion
NM_000878	Interleukin 2 receptor, beta	IL2RB	−1.158	Cytokine-mediated signaling pathway // positive regulation of survival gene product expression
NM_006850	Interleukin 24	IL24	−1.106	Apoptosis
NM_001562	Interleukin 18 (Interferon-gamma-inducing factor)	IL18	−1.045	Angiogenesis // immune response // positive regulation of interferon-gamma production
NM_000576	Interleukin 1, beta	IL1B	−1.024	Positive regulation of protein phosphorylation // inflammatory response // signal transduction
**NM_002188**	**Interleukin 13**	**IL13**	**−0.550**	**Inflammatory response // cell-cell signaling // positive regulation of protein secretion**

Source: [31].

**Table 2:** List of PCR primer sequences.

Primer Names	Primer Sequences (5′ to 3′)		Products Size (bp)
**GAPDH mRNA**	Forward	TGGTATCGTGGAAGGACTCATGAC	189
	Reverse	ATGCCAGTGAGCTTCCCGTTCAGC	
**IL-13 mRNA**	Forward	CATGGCGCTTTTGTTGACCA	181
	Reverse	AGCTGTCAGGTTGATGCTCC	
**IL-13Rα1 mRNA**	Forward	CATGAAGAGGATGCTGTGAA	172
	Reverse	TAGGGATCACAACCACCACA	
**IL-13Rα2 mRNA**	Forward	ATATTTACTCTGTTCTTGGA	132
	Reverse	ATATTTTGTCCATCAGCCTT	
**IL-13 (ChIP: STAT3/6) #1**	Forward	AAACGAGGGAAGAGCAGGAA	143
	Reverse	CCCTTTGCTCACCAGTCTCT	
**IL-13 (ChIP: STAT3/6) #2**	Forward	TGGCACTAGGAACACTTACA	136
	Reverse	AGAGTGGCTGGAAGTAGTGT	
**IL-13 (ChIP: STAT3/6) #3**	Forward	TTT ACT CTT CTA TTG CCT GT	104
	Reverse	TAT GGG AAT TTG GGG AGT TT	
**IL-13Rα2 (ChIP: STAT3/6) #1**	Forward	TGGGTTAGTGGCATTGAAAG	109
	Reverse	AAAAAAAACCTCCAGGAAGT	
**IL-13Rα2 (ChIP: STAT3/6) #2**	Forward	GAAAACACTGGCATACTAAG	132
	Reverse	TGCATTCAAAATAGGTCAGC	

## Supplementary Materials

Supplementary materials can be found at https://www.mdpi.com/1422-0067/20/8/1949/s1.

## Abbreviations

CCL5	Chemokine (C-C motif) ligand 5
IL-4Rα	Interleukin-4 receptor subunit alpha
IL-13Rα1	Interleukin-13 receptor subunit alpha 1
JAK	Janus kinase
MAPK	Mitogen-activated protein kinase
NF-κB	Nuclear factor kappa light chain enhancer of activated B cells
PCR	Polymerase chain reaction
STAT	Signal transducer and activator of transcription
TNF-α	Tumor necrosis factor alpha
TYK	Tyrosine kinase
