# Supplementary material for: Inhibition of IL-13 and IL-13Rα2 Expression by IL-32θ in Human Monocytic Cells Requires PKCδ and STAT3 Association
Source: Int J Mol Sci. 2019 Apr 20;20(8):1949. doi: 10.3390/ijms20081949 (PMC6514684; doi:10.3390/ijms20081949)
Supplement: Supplementary file 1 [file ijms-20-01949-s001.pdf]

Supplementary Materials

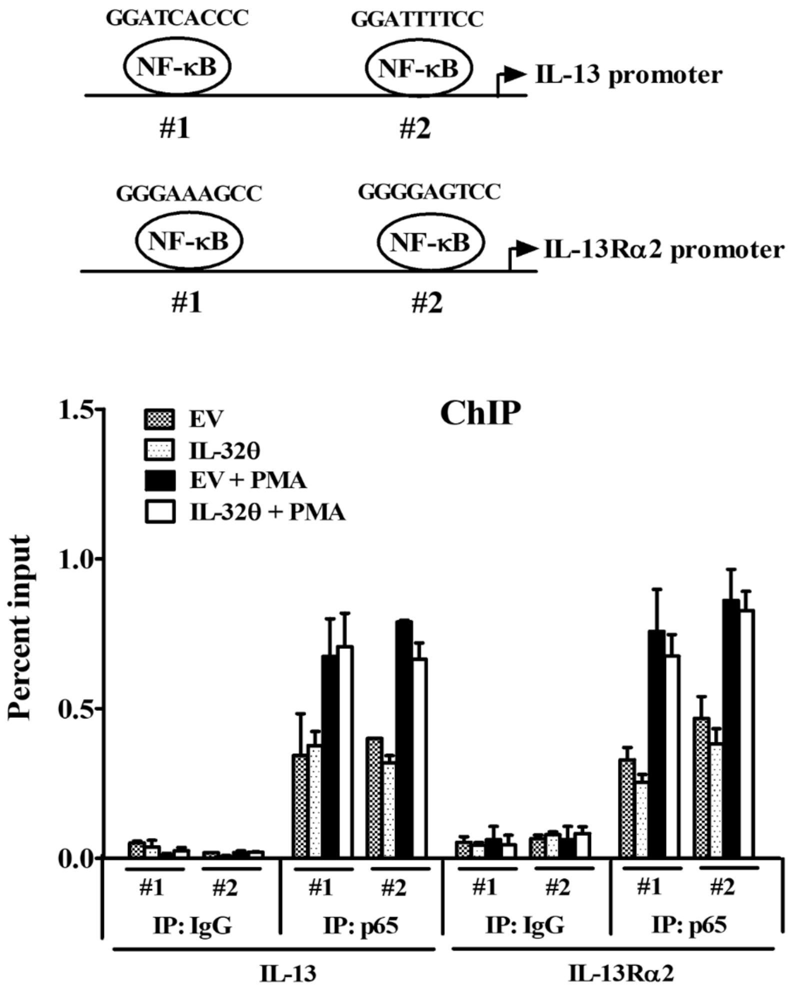

Supplementary Figure S1. The NF-κB binding sites of IL-13 or IL-13Rα2 promoters were not mediated by IL-32θ. Up panel, diagram showing putative element sites for NF-κB binding to IL-13 or IL-13Rα2 promoters. Down panel, ChIP assay (n = 3) was conducted by performing immunoprecipitation of p65, an NF-κB subunit, followed by quantitative PCR using specific primers to target 2 binding sites of p65 in the IL-13 promoter and 2 binding sites in the IL-13Rα2 promoter. Data are shown as mean ± SEM. Statistical significance was analyzed using two-way ANOVA test followed by multiple comparison tests. The results are not statistically significant.

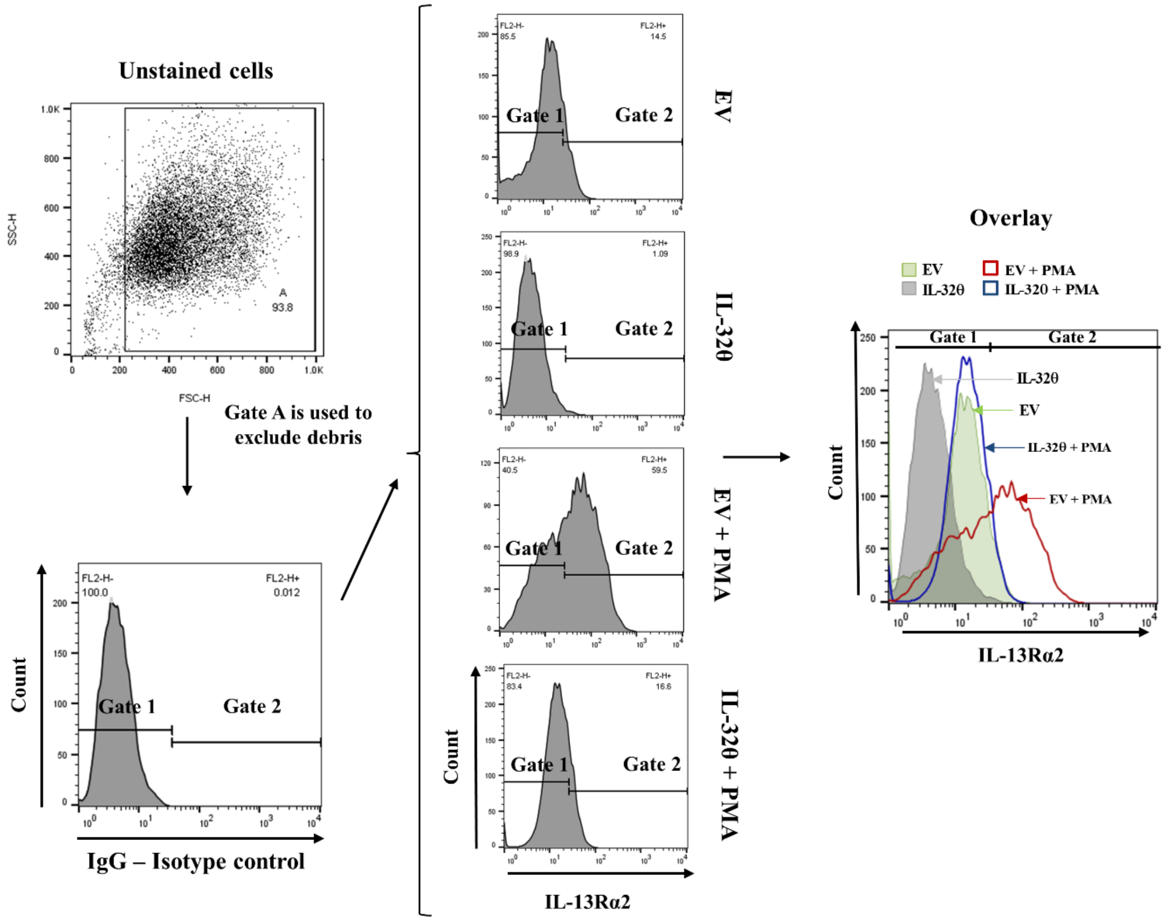

Supplementary Figure S2. Flow cytometry analysis gating strategy. First, cell populations were distinguished based on their forward and side scatter properties. Since THP-1 cells are monocytic cells, there is only one population of cells. Gate A was created to exclude debris and dead cells. Second, the cells within the Gate A can be further analyzed for isotype control expression by single parameter histogram. Based on the histogram of isotype control, two gates were created: Gate 1 contains 100% of cells in isotype control peak area, reflecting the cells which do not express IL-13Rα2, gate 2 contains the rest of the cells, reflecting the cells which express IL-13Rα2. Third, the Gate1/Gate 2 system was applied to each group of cells separately (EV, IL-32θ, EV+PMA, IL-32θ+PMA), and then the quantification was performed. Fourth, the overlay image is created as a representative image containing all histogram peaks of one set of experiment.
